# Research on the International Sustainable Practice of the Taiwanese Food and Agricultural Education Law under the Current Global Food Security Challenges

**DOI:** 10.3390/foods12142785

**Published:** 2023-07-21

**Authors:** Chih-Cheng Huang, Shang-Pin Li, Jiin-Chyuan Mark Lai, Yung-Kuan Chan, Ming-Yuan Hsieh

**Affiliations:** 1 Department of Leisure & Recreation, National Formosa University, Yunlin City 403632, Taiwan; cchuang@nfu.edu.tw; 2 Department of Early Childhood Development and Education, Chaoyang University of Technology, Taichung City 41349, Taiwan; spli@cyut.edu.tw; 3 Department of Applied Foreign Languages, TransWorld University, Yunlin City 403632, Taiwan; marklai07@gmail.com; 4 Department of Management Information Science, National Chung Hsing University, Taichung City 402204, Taiwan; 5 Department of International Business, National Taichung University of Education, Taichung City 403454, Taiwan

**Keywords:** international sustainable compatibility, food health and agriculture safety (FHAS), Taiwanese Food and Agricultural Education Law (TFAEL), institutional theory (IT), learning community model (LCM), social learning theory (SLT)

## Abstract

On 19 April 2022, in order to overcome a succession of recent global food health and agriculture safety (FHAS) incidents, the Taiwanese government instituted and announced 20 comprehensive Articles under the Taiwanese Food and Agricultural Education Law (TFAEL) for regulating the Taiwanese FHAS in order to completely construct the international strategies under the current global food security challenges of the FAHS. As a result, this research study firstly employs the learning community model (LCM) of the learning theory to measure the implementing depth of the international sustainability practice of the 20 Articles of the Taiwanese FHASE from a learning performance analytical perspective. Then, the social learning theory (SLT) of the educational theory is applied to consolidate the individual behaviors of the relative stakeholders, the organizational management of the relative industries and the socialized consent of the multilateral organization performance assessment network (MOPAN) of the Food and Agriculture Organization (FAO) of the United Nations (UN). After completing quantitative and qualitative analyses, the two most valuable conclusions and findings were as follows: (1) Not only Article 12, but also Articles 3, 4 and 5 of the new law can empirically facilitate strategic management for supporting and promoting the Taiwanese FHAS educational policy dialogue at a global, regional and country scale in order to effectively advance the international sustainable compatibility of the TFAEL. (2) Articles 6, 11 and 16 of the new law can completely construct operational management for building normative and standard-setting Taiwanese FHAS educational works in order to efficiently advance the international sustainable compatibility of the TFAEL. Therefore, beyond the analytical results of this research, the international sustainable compatibility of the TFAEL provides a direction for the development of sustainable food systems, food policies, agricultural food markets and food chains in order to overcome the various contemporary global food security challenges.

## 1. Introduction

For a long time, governments have paid attention to stabilizing the supply and volatility of food prices and crops, and to technical guidance and marketing promotion on the agricultural production side. Further, most people have deemed that the food health and agriculture safety (“FHAS”) issue is the government’s responsibility [1], which resulted in a succession of recent food safety incidents. These incidents include the FHAS issue of contaminated eggs that broke out in Europe on 1 August 2017; the exposure of mad cow disease in North America in 2017; the African swine fever outbreak in 2018 and so forth. Additionally, traditional agricultural promotion often focuses on technical guidance and marketing promotion on the agricultural production side, and the people have taken a stony silence regarding the public FHAS incidents. Consequently, according to the information released by the World Health Organization (WHO) in April 2022, global food security challenges have caused up to 600 million people to suffer from diseases resulting from FHAS incidents around the world. Significantly, this wave has also led each nation’s government to commence the construction of a series of regulations and rules around FHAS [2]. Only high-quality ingredients and food can produce delicious, nutritious and pollution-free dishes to the public. Particularly, FHAS is related to the overall security lifeline of the country [3], and for this reason, each government has adopted various educational methods to improve people’s concepts and practices with regard to global food security challenges [4]. Recently, the FHAS sustainable development goal was issued in the 2014 official report of the multilateral organization performance assessment network (MOPAN) of the Food and Agriculture Organization (FAO) of the United Nations (UN). This report provides an assessment of four dimensions for reviewing the region’s development results, performances and snapshots in FHAS in order to regulate individual behaviors and public consent through surveys in six countries. These four assessed dimensions are the following: (1) the strategic management for supporting and promoting the TFHAS policy dialogue at a global, regional and country scale (“SM-SPPDGRCL”); (2) operational management for building normative and standard-setting TFHAS works (“OM-BNSW”); (3) relationship management for facilitating TFHAS advocacy, communication and sharing of data and information (“RM-FACSDI”); and (4) management for encouraging TFHAS knowledge and the uptake and updating of technology (“KM-EKTUU”). These four assessed dimensions can analyze the cross-measurements of the role of cognitive institutions [5].

Recent incidents of the Taiwanese food health and agriculture safety (“TFHAS”) include the contaminated soy sauce and starch food safety incident in 2013, the rancid cooking and feed oil that was used as edible oil in 2014, the “Min Dou” pesticide residue food safety incident of schools’ nutritious lunches in 2014, the pesticide residues in tea beverages in hand-shaken beverage shops in 2012 and 2015, the presence of antibiotics in honey in 2015, the high fipronil content in eggs in 2017, the pudding powder and other products made with expired additives in 2018, and in 2019, a high fipronil content was once again detected in eggs. Therefore, the Taiwanese public has started to break their silence by expressing their own opinions on FHAS. They strongly request more TFHAS regulations and rules in nutrition, housekeeping, health, food health, agriculture safety and ecological education. In order to effectively reflect these public requirements, the Taiwanese government has specially integrated not only the TFHAS standards, but also educational concepts and behaviors into the legislation source design of the 20 Articles of the Taiwanese Food and Agricultural Education Law (“TFAEL”) on 19 April 2022 to overcome the current global food security challenges. Firstly, the TFAEL has officially declared the Executive Yuan, at the Council of Agriculture, as the main authority responsible for the entire TFHAS. Secondly, new goals and principles were incorporated into the TFAEL in order to bring positive benefits to the expansion of domestic ingredient use for local production and public consumption [6,7,8].

However, it is difficult to make a comprehensive survey on the international sustainable compatibility of the TFAEL [9,10,11] after the announcement, institution and administration of the 20 Articles because there is no professional academic journal paper that is able to effectively and thoroughly assess the international sustainable compatibility of the TFAEL under the various global food security challenges. Therefore, the institutional theory (IT) [12] was employed for considering the international convergence of legislation to explain and understand the phenomenon of legal convergence in order to systematically explore and evaluate the international sustainable compatibility of the TFAEL, because the institutional theory (IT) was designed to emphasize the normative role of legal systems in shaping behavior. In the context of research on the international convergence of legislation, the institutional theory (IT) can be used to analyze the similarities and differences between legal systems of different countries, as well as to analyze the impact of legal institutions on international convergence through institutional norms and constraints. This theory explores the role of normative, regulative and cognitive institutions of the TFAEL in shaping legal convergence in the developed tendency of the TFHAS.

**Figure 1:**
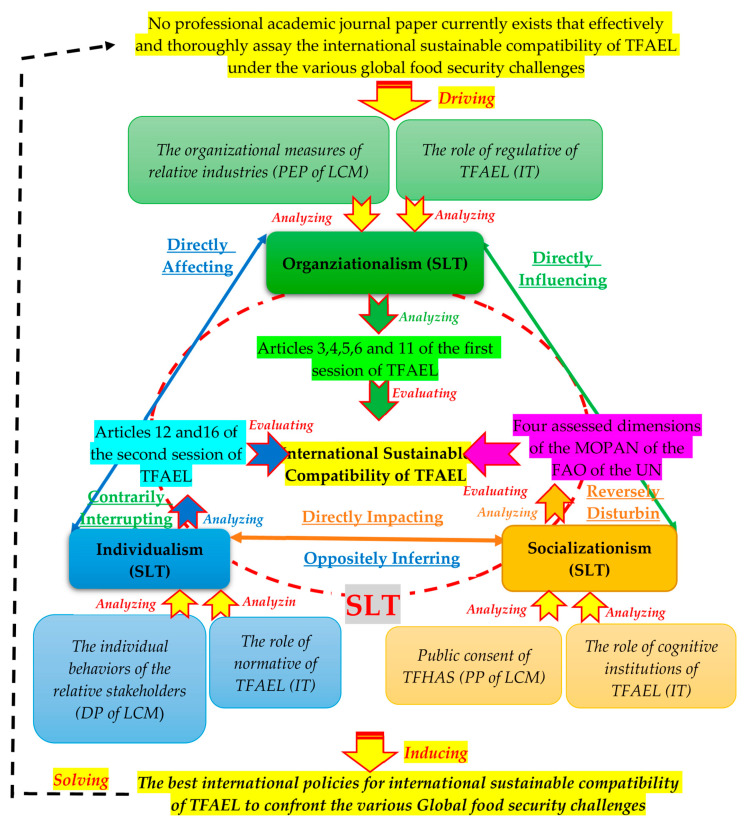
Main research conceptual framework.

In order to extensively consider the educational sustainability of these 20 Articles of the TFAEL, the public, democracy and pursuing excellence philosophies of the learning community model (LCM) [13] from the learning theory were employed to assess the legal educational performance and effectiveness of the TFHAS on the entire Taiwanese society. This is because the TFHAS is not only a law to establish a legal basis, to deepen the understanding of the TFHAS among people of different age groups such as families, schools and communities and to promote the effective combination of catering the TFHAS affairs, but it is also a non-stopped educational process that encourages citizens to pay more attention to their own food health and the sustainable development of farming and fishing villages, agriculture and the environment, and to take real actions. Eventually, the social learning theory (SLT) [14] of the educational theory was applied to evaluate the TFAEL roles of normative, regulative and cognitive institutions of the institutional theory (IT) and the public, democracy and pursuing excellence philosophies of the learning community model (LCM) were used to comprehensively appraise the interactive dependency and relations from the three basic assessed aspects (individual behaviors—individualism; organizational measures—organizationism; public consent—socializationism) in order to induce the best international policies for the international sustainable compatibility of the TFAEL to confront the various global food security challenges, as described in Figure 1.

In Figure 1, the public, democracy and pursuing excellence philosophies of the learning community model (LCM) were able to measure the individual behaviors of the relative stakeholders, organizational measures of relative industries and public consent of the TFHAS [15] in the international sustainable compatibility of the TFAEL. The reasons for this are as follows: (1) The TFHAS educational circumstance can be considered as a big learning stakeholder community, including each TFHAS-related competent authority, responsible agency or department, local organization, relative industry and each Taiwanese person [16]. (2) Significantly, there are some inseparable relationships in this big learning stakeholder community. (3) Continuously, each Taiwanese person is supposed to respect the TFAEL in order to effectively improve and efficiently protect the World Health Organization’s living environment. (4) Ultimately, each Taiwanese person is also responsible for advancing the TFHAS [17]. Finally, the social learning theory (SLT) [18] recognizes that the relationships and reciprocal influences among individuals, organizations and society are reciprocal and dynamic because individual concepts and behaviors shape organizational practices through a succession of learning processes, while organizations influence individual behavior through rewards, punishments and socialization processes. At the same time, societal changes and tendencies influence both individuals and organizations, driving adaptations and learning processes. Specifically, each individual is the most important and essential element of organizations and societies. Therefore, positive and negative mutual influences indeed exist among individuals, organizations and society.

In order to statistically measure the interplays, the factor analysis (FA) [19] approach of quantitative analysis was adopted to measure the questionnaire weights of the assessed criteria of the individual behaviors of the relative stakeholders, the organizational measures of relative industries and the TFHAS public consent on the international sustainable compatibility of the TFAEL, because the FA of quantitative analysis was created to systematically measure communalities (dependences) by administering large-scale questionnaires with a higher research validity and representativeness. Then, in light of the measurement of the interactive dependences among the individual behaviors of the relative stakeholders, the organizational measures of relative industries and the TFHAS public consent in the international sustainable compatibility of the TFAEL, the triangle entropy method (TEM) [20] of qualitative analysis was employed to develop the international sustainable compatibility of the TFAEL for overcoming the contemporary various global food security challenges with a higher research reliability and accuracy.

## 2. Review of the Literature 

### 2.1. Demonstration of Main Concepts and Assessed Theories

#### 2.1.1. The Content of the TFAEL

Significantly, the 20 Articles of the TFAEL can be refined based on [8].

(1) First session of the TFAEL:

1. Articles 1 to 4 legislatively institute the power and responsibility of the TFHAS to the relative competent authorities to effectively facilitate the participation of the Taiwanese public in promoting the developed sustainability of the TFAHS.
2. Article 3 firstly defines the empirical implemented terms of the educational activities of the TFAHS. Its content is described as follows: “TFHAS is to refer to the use of educational methods to cultivate citizens’ understanding of national basic agricultural production, agricultural product processing, friendly environment, friendly production and breeding and animal husbandry, animal welfare, food selection, knowledge and practice of catering preparation, leftover food disposal, and enhance the connection between food, the environment and agriculture” (TFAEL, 2022).
3. Articles 4, 5 and 6 legally point out that each level of the central, municipal and local Taiwanese government must set the TFHAS-related competent authorities, responsible agencies or departments to facilitate the planning, supervision, awards and professional labor training of the TFHAS. Furthermore, these TFHAS-related competent authorities have the direct duty to organize professional committees to advance the implementation, performance, development and gathering resources for the administrative guidance of the TFHAS.
4. Article 5 directs each TFHAS-related competent authority, responsible agency or department to take the legal rights and responsibilities to dominate the direction of international development and social needs, formulate promotion policies and set up specific implementation indicators for comprehensively developing sustainability.
5. Article 8 states that each TFHAS-related competent authority, responsible agency or department has to regularly hold two meetings per year to update the amendment-related policies by inviting various experts, scholars and non-governmental representatives.
6. Article 11 directly empowers each TFHAS-related competent authority, responsible agency or department to have the legal priority to expropriate local agricultural products or foods to all national government agencies (departments), public institutions, administrative legal persons, schools, kindergartens and foundations in order to sustainably develop TFHAS-related industries.

In order to improve the labeling regarding the place of production or origin, Articles 12 and 16 currently regulate that primary food staples consumed by Taiwanese people such as egg, rice, chicken, soybean, etc., must be clearly labeled to identify the origins of these primary food and agricultural products.

(2) Second session of the TFAEL:

1. Article 12 regulates that each TFHAS-related competent authority, responsible agency or department has to guide cater and food businesses to use local ingredients; label the origin of the ingredients according to the municipality, county (city) or township (town, city) name; promote the consumption of local agricultural products; reduce food waste; reduce the amount of ingredients and reduce leftovers. Thus, Article 12 not only effectively encourages food and catering industrialists to use local ingredients while labeling the original production location and to reduce leftovers in the operation, but it also efficiently guides consumers to think about the sources of necessary ingredients by giving priority to domestic and local ingredients sourced in Taiwan.
2. Article 16 regulates that each TFHAS-related competent authority, responsible agency or department has to set up not only a network platform to integrate relevant information, but to also construct a food and agriculture education information integration platform to strengthen consumption channel information, promote local agricultural products and labels, and integrate food and agriculture education teaching materials, teaching plans, professionals, teachers, volunteers and publicity materials.
3. Articles 12 and 16 manifestly rule that each TFHAS-related competent authority, responsible agency or department has to design and offer the appropriate rewards to the TFHAS-related industrialists and institutions that provide outstanding contributions.

As Taiwan is a part of the global economy, these 20 Articles of the TFAEL must not only be considered, but they must also be measurable in order to comprehensively develop the internationalization and sustainability of the TFHAS, because the international sustainable compatibility can effectively advance the legitimacy, effectiveness, implemented performance and international sustainability of the TFAEL through the international sustainable compatibility.

#### 2.1.2. The Institutional Theory (IT)

In order to achieve the research goal, the institutional theory (IT) was applied to emphasize the impact of institutions on the international convergence of the TFHAS. It can be argued that the TFHAS system and the TFAEL rules shape the country’s overall individual behaviors and organizational cooperation model and drive all Taiwanese people to adopt and abide by common legal standards through institutional norms and constraints. The institutional theory (IT) originally emerged in the late 19th and early 20th centuries to be formed as a prominent sociological perspective used to examine the role of institutions and society in shaping individual and collective behavior. The core concepts were (1) the significance and role of normative, regulative and cognitive institutions in regulating individual behaviors and organizational policies, and (2) the role of rational–legal authority and formal organizations in society. Comprehensively, the institutional theory (IT) continues to evolve, incorporating insights from other disciplines such as economics and political science. It has expanded to study fields beyond organizations, including international institutions, healthcare, education and sustainability. Continuously, a lot of researchers began to utilize the institutional theory (IT) to examine how multiple institutional logics or socially constructed systems of values, norms and beliefs coexist and influence organizations and society. This perspective emphasizes the tension and conflicts that arise when individuals and organizations are subjected to different institutional pressures. Overall, the institutional theory (IT) can not only evolve, incorporating insights from other disciplines such as economics and political science, but it can also be used to extensively study fields beyond organizations, including international institutions, healthcare, education and sustainability.

#### 2.1.3. The Learning Community Model (LCM)

In order to effectively advance the legitimacy, effectiveness, implemented performance and international sustainability of the TFAEL, the democracy philosophy (DP), pursuing excellence philosophy (PEP) and public philosophy (PP) of the learning community model (LCM) have further been employed to assess the legal educational performance and effectiveness of the TFHAS on the entire Taiwanese society, as shown in Figure 1.

In detail, firstly, the democracy philosophy (DP) of the learning community model (LCM) [21] can demonstrate that most Taiwanese people consent to the international sustainable compatibility of the TFHAS. The reason for this is that the TFAEL offers the equal right of each Taiwanese person to speak and attend various TFHAS activities and political campaigns to practically improve food safety and promote ecological conservation in the international sustainable compatibility of the TFHAS from the normative considered role of the institutional theory (IT) in the TFAEL.

Secondly, the pursuing excellence philosophy (PEP) of the learning community model (LCM) [21] can illustrate that the TFAEL is a strong and clear regulation to encourage each TFHAS-related competent authority, responsible agency or department, local organization, relative industry and Taiwanese person to support the implementation of the various TFHAS activities and political campaigns for the entire Taiwanese society from the regulative considered role of the institutional theory (IT) in the TFAEL. Specifically, the organizationism characteristic of the SLT can measure the PEP aspect of the LCM and cognitive institutions’ considered role of the institutional theory (IT) in the TFAEL at the same time. The reason for this is that each TFHAS-related competent authority, responsible agency or department has the authorities and duties to administer and promote the TFAEL performance in order to facilitate the TFHAS for the entire Taiwanese society.

Thirdly, the public philosophy (PP) of the learning community model (LCM) [21] can present that the TFAEL is a legal standard regarding the educational activities and measures of the TFHAS. The TFAEL can provide public legal sources, financial grants and high-quality resources to support the international sustainable compatibility of the TFHAS for the entire Taiwanese society from the cognitive institutions’ considered role of the institutional theory (IT) in the TFAEL. Particularly, the socializationism characteristic of the SLT was consolidated into the public philosophy (PP) of the learning community model (LCM). The reason for this is that the entire Taiwanese society has to endure the implementation outcomes and results of the TFHAS by completely respecting the TFAEL to confront the various global food security challenges.

#### 2.1.4. The Social Learning Theory (SLT)

**Figure 2:**
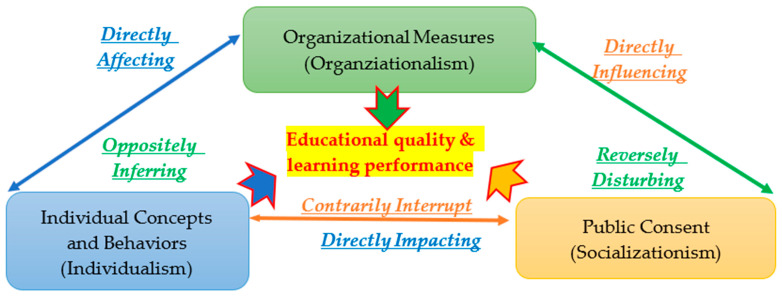
Three main characteristics of the SLT.

In order to comprehensively assay the interactions and dependences among the democracy philosophy (DP), pursuing excellence philosophy (PEP) and public philosophy (PP) of the learning community model (LCM), the social learning theory (SLT) was further applied, as shown in Figure 1. The social learning theory (SLT) was applied to an in-depth exploration on the interplays among the three brief philosophies that were created and advocated in the learning community model (LCM), and the TFAEL roles of the normative, regulative and cognitive institutions of the institutional theory (IT) were consolidated from three basic evaluated characteristics (individual concepts and behaviors—individualism; organizational measures—organizationalism; public consent—socializationism) [22,23], as illustrated in Figure 2.

Significantly, the individualism characteristic of the social learning theory (SLT) can explore both the democracy philosophy (DP) of the learning community model (LCM) and the normative considered role of the institutional theory (IT) in the TFAEL. Each Taiwanese citizen has equal rights and duties to speak and attend the various TFHAS activities and political campaigns. Particularly, the individual concepts and behaviors of the TFHAS-related stakeholders (individualism) directly affect the organizational measures of the TFHAS-related industries (organizationalism) [24]. Reciprocally, the organizational measures of the TFHAS-related industries (organizationalism) can also oppositely infer the individual concepts and behaviors of the TFHAS-related stakeholders (individualism) that directly influence the public consent of the TFHAS (socializationism) regarding the multilateral organization performance assessment network (MOPAN) of the Food and Agriculture Organization (FAO) of the United Nations (UN) [25].

At the same time, the TFHAS public consent (socializationism) regarding the multilateral organization performance assessment network (MOPAN) of the Food and Agriculture Organization (FAO) of the United Nations (UN) can also reversely disturb the organizational measures of the TFHAS-related industries (organizationalism). Critically, the TFHAS public consent of the TFHAS (socializationism) regarding the multilateral organization performance assessment network (MOPAN) of the Food and Agriculture Organization (FAO) of the United Nations (UN) can also directly impact the individual concepts and behaviors of the TFHAS-related stakeholders (individualism) and the organizational measures of the TFHAS-related industries (organizationalism) [26]. Meanwhile, the individual concepts and behaviors of the TFHAS-related stakeholders (individualism) and organizational measures of the TFHAS-related industries (organizationalism) can also contrarily interrupt the public consent of the TFHAS (socializationism) regarding the multilateral organization performance assessment network (MOPAN) of the Food and Agriculture Organization (FAO) of the United Nations (UN) [27].

Hence, the officers and staffs of the TFHAS-related competent authorities have to accomplish a series of relative classes of the FHASE and then must report the completed learning outcomes and performances in connection with the international sustainability practice. Measurably, the first session of Articles 3, 4, 5, 6 and 11 were defined as the pursuing excellence philosophy (PEP) of the learning community model (LCM) for assessing the organizational management of TFHASE-related industries and the second the Article 12 and 16 were classified as the democracy philosophy (DP) of the learning community model (LCM) [28] for appraising the individual behaviors of the TFHASE-related stakeholders.

### 2.2. Illustration of Evaluated Methods

#### 2.2.1. The Factor Analysis (FA) of Quantitative Analysis

In terms of the achievement of research reliability, representativeness, validity and accuracy, this research cross-employs the factor analysis (FA) of quantitative analysis [29] to condition the measurement of a large-scale questionnaire as well as the triangle entropy method (TEM) of qualitative analysis [30] to deal with the weighted assessment of an expert’s questionnaire. Historically, the factor analysis (FA) of quantitative analysis was created to detect, identify and classify the interactive dependences and categorization of evaluated factors through a series of weighted compared computations. The brief computation of the factor analysis (FA) of quantitative analysis measured the relation weights between the independent variables (direct unobserved influenced factors) presented as $X\left(X_{1},X_{2},\ldots ,X_{k}\right)$ and the dependent variables (direct observed influenced factors) defined as $Y\left(Y_{1},Y_{2},\ldots ,Y_{k}\right)$. Equation (1) of the factor analysis (FA) of quantitative analysis was discussed as the relation between two variables [31,32,33].

$$X_{1}=\lambda_{11}Y_{1}+\lambda_{12}Y_{2}+\cdots +\lambda_{1k}Y_{k}$$

$$Y_{\_}=P^{1}X_{\_}\;,\;X_{\_}=P^{1}Y_{\_}$$

Step 1: Standardize the intersection of variance to be 1 (maximum).

If maximization occurs:

$$X_{k}-u_{k}=\lambda_{k1}f_{1}+\lambda_{k2}f_{2}+\cdots +\lambda_{km}f_{m}+e_{k}$$

(s.t.${\left(X-u\right)}_{-}{}_{k*1}=\wedge_{m}{}_{k*m}f_{m*1}+e_{-}{}_{k*1}$), variance–covariance matrix presents as

$$\sum ={\Lambda \Phi \Lambda }^{1}+\Psi ,\;\Psi =diag\left(\Psi_{1},\Psi_{2},\ldots ,\Psi_{m}\right)$$

(1) $$\left(s.t.\;\phi =I_{m*m}\;\right)$$

#### 2.2.2. The Triangle Entropy Method (TEM) of Qualitative Analysis

In order to increase the research reliability, accuracy and professionalism, the “discrete probability connections” in the interaction-compared assessments of each evaluated criterion $\left(P_{1},P_{2},\ldots ,P_{k}\right)$ was created in Equation (2) of the assessment of the entropy method, as performed in [34,35,36].

(2) $$E\left(P_{1},P_{2},\ldots ,P_{k}\right)=-\phi_{k}\overset{k}{\underset{i=1}{\sum }}P_{i}*In\left(P_{i}\right)$$

Step 1: $\phi_{k}=1/I\left(k\right)$ was the normal quantity and $O\leq E(P_{1},P_{2},\ldots ,P_{3)}\leq 1$.

Step 2: The number of $E\left(P_{1},P_{2},\ldots ,P_{k}\right)$ was reversely associated with the interactive dependences among each assessed criterion.

In detail, in the extension of Equation (2), the interaction-compared measurements of the “discrete probability connections” in the interactive dependences were completely computed in the entropy triangular weight ($H\left(\frac{Y}{X}\right)$) measurements based on Equation (2), as described in Equation (3) from [37].

(3) $$H\;\left(\frac{Y}{X}\right)=\underset{x\in X}{\sum }P\left(x\right)*H(\frac{Y}{X}=x)\;=-\underset{x\in X}{\sum }P\left(x\right)*p(\frac{Y}{x})*logP\left(y/x\right)\;=-\underset{x\in X,y\in Y}{\sum }P\left(x,y\right)*p(\frac{Y}{x})\;=-\underset{x\in X,y\in Y}{\sum }P\left(x,y\right)*logP\left(y/x\right)\;=-\underset{x\in X,y\in Y}{\sum }P\left(x,y\right)*log(\frac{P\left(\frac{y}{x}\right)}{P\left(x\right)})\;=\underset{x\in X,y\in Y}{\sum }P\left(x,y\right)*log(\frac{P\left(x\right)}{P\left(\frac{y}{x}\right)})$$

Significantly, the interplays and dependences among the individual behaviors of the TFHAS-related stakeholders, organizational management of the TFHAS-related industries and TFHAS socialized consent of the multilateral organization performance assessment network (MOPAN) of the Food and Agriculture Organization (FAO) of the United Nations (UN) were comprehensively assessed by systematically analyzing the structure of the evaluated mode of the international sustainable compatibility of the TFHAS through the effective assessments of the triangle entropy method (TEM) of qualitative analysis.

## 3. Research Design

### 3.1. Research Steps

**Figure 3:**
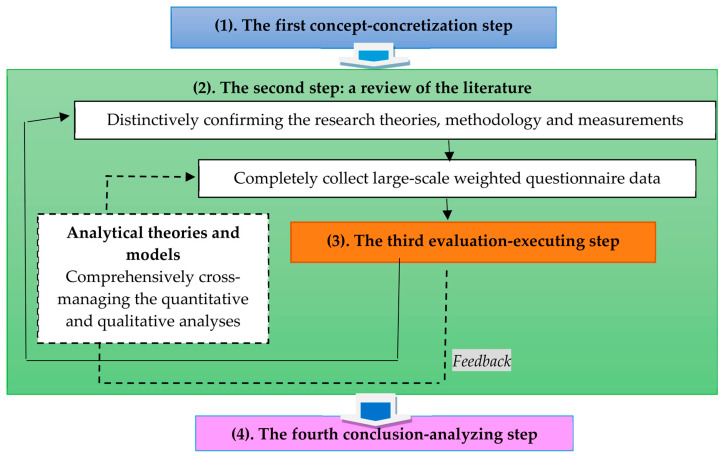
Four research steps.

As shown in Figure 1 and Figure 2, the most core determinants of the international sustainable compatibility of the TFAEL under the current global food security challenges are going to be induced to achieve the main goal of this research using the four research steps shown in Figure 3.

In Figure 3, the concrete actions of the four research steps are described as follows:

(1) The first step: We concretely explored the most core determinants of the international sustainable compatibility of the TFAEL under the current global food security challenges to achieve the main goal of this research by cross-employing the institutional, learning community and social learning theories and statistical analyses of the factor analysis (FA) and triangle entropy method (TEM).
(2) The second step: We not only extensively explored the literature about the main concept of the FAHS and TFAEL, but also systematically reviewed the literature about the institutional, learning community and social learning theories as well as comprehensively integrated the statistical analyses of the factor analysis (FA) and triangle entropy method (TEM).
(3) The third step: We implemented the factor analysis (FA) of quantitative analysis in detail to empirically deal with the large-scale questionnaire data with a higher research validity and representativeness, as well as used the triangle entropy method (TEM) of qualitative analysis to practically execute the expert’s weighted questionnaire results with a higher research reliability and exactness.
(4) The fourth step: Based on the systematic measurements and assessments of the statistical analyses of the factor analysis (FA) and triangle entropy method (TEM), we induced the valuable conclusions and contributive findings in order to academically resupply the interdisciplinary research gap and empirically provide the most effective recommendations for the international sustainable practice of the Taiwanese Food and Agricultural Education Law under the current global food security challenges.

### 3.2. Evaluated Criteria

According to Figure 1 and Figure 2, there were three essential appraised, assessed and evaluated criteria to be analyzed in depth with respect to the literature. Articles 12 and 16 of the TFASEL were the two evaluated criteria in the first appraised criteria group used to analyze the cross-measurements of the normative role in regulating individual behaviors and organizational policies and individual behaviors of the TFHAS-related stakeholders (individualism) in the international sustainable compatibility of the TFAEL. Articles 3, 4, 5, 6 and 11 of the TFASEL were the five assessed criteria in the second appraised criteria group used to measure the role of regulating individual behaviors and organizational policies and organizational measures of the TFHAS-related industries (organizationalism) in the international sustainable compatibility of the TFAEL. The four assessed dimensions (SM-SPPDGRCL, OM-BNSW, RM-FACSDI and KM-EKTUU) of the multilateral organization performance assessment network (MOPAN) of the Food and Agriculture Organization (FAO) of the United Nations (UN) were defined as the evaluated criteria in the third appraised criteria group.

### 3.3. Questionnaire Content

With reference to the methodological measures of the factor analysis (FA) of quantitative analysis [38], the content of the first questionnaire was designed to ask the participants how much importance they consider the SM-SPPDGRCL to have in the international sustainable compatibility of the TFAEL [39]. The contents of other questionnaires from the second to the eleventh were also designed using the contents of the first questionnaire. Subsequently, the content of the questionnaire for the expert’s pairwise comparison matrix in the methodological measures of the triangle entropy method (TEM) of qualitative analysis was designed to ask the participants how much importance they believe the SM-SPPDGRCL has compared to the OM-BNSW for the international sustainable compatibility of the TFAEL considering Article 3. In detail, the 5-point Likert’s scale was utilized in the methodological measures of the factor analysis (FA) of quantitative analysis and the triangle entropy method (TEM) of qualitative analysis. Finally, the questions were instituted as the eleven questions, which is the same quantity as the eleven assessed criteria.

### 3.4. Collected Questionnaires

Essentially, the larger the number of samples selected, the higher the accuracy of the inference of the statistical analysis results to the actual situation, which can help to avoid the effective detection of differences caused by too few samples. Significantly, the authors of [40] addressed the interviewed quantity of up to 250 and the valid rate of up to 90% (231) for the highest research reliability in the factor analysis (FA) of quantitative analysis. Furthermore, according to the academic ethic regulations in society science research and academic ethic policies of the Taiwanese Ministry of Science and Technology Council and Ministry of Education, the large-scale collected questionnaires of this research have conformed the five conditions for the censorship free of society science research. Therefore, first of all, the survey sample of this research was settled with a quantity of 250, and all participants were adults who are older than 18 years of age. Secondly, only the questionnaire fulfillment method was utilized without any intrusive methods in the collected procedure. Thirdly, there was no personal information and identified characteristics to be displayed in the questionnaire content and research. Fourthly, these 250 interviewees totally realized the entire research goal and processes. Fifthly, the questionnaire collection process was conducted without any intrusive survey measures. Finally, the 231 valid questionnaires were collected in person from the 250 related industrialists in the TFAHS, and the regions of these valid interviewees were determined via simple random sampling. The other 19 declined to participate in this research. Critically, they consented for the surveyed questionnaires to be used in the Equations (1) and (2) of the factor analysis (FA) of quantitative analysis for this research. Hence, in view of higher research validity and representativeness, the original data size had to be limited to a total of the 250 industrialists in the food and agriculture relative fields, and eventually, of this total, 231 valid questionnaires were considered to be valid and accepted for this research. The valid retrieval of these weighted questionnaires is 92.4%, and in detail, the analytical description is expressed in Table 1.

In detail, there were 199 interviewees that had previously taken related courses regarding the international sustainable compatibility of the TFHAS. This induces that approximately 86.15% of the interviewees have experience with regard to the international sustainable compatibility of the TFHAS. Fifty-seven (57) out of the 231 interviewees (or 24.68%) were not willing to support the international sustainable compatibility of TFHAS, even if they were knowledgeable on the contents of Article 5 of the TFAEL. In addition, with respect to the expert’s questionnaires, [41] clearly induced that the experts’ and professionals’ collected questionnaires have to up to at least over 10 percent of the entire large-scale surveyed data in order to establish the least errors of higher research validity and reliability in the data collection. For this reason, there were 30 experts and professionals who were designed to be interviewed in person for obtaining the experts’ evaluated measurements using the triangle entropy method (TEM) of qualitative analysis in order to practically explore the level of the internationalization and developed sustainability of the TFAEL. In detail, the first 10 experts have over ten years of academic research in the TFHAS-related research fields. The other 10 experts have over ten years of working experience in the TFHAS-related industries. Finally, the other 10 experts came from the TFHAS-related departments of the Taiwanese government with over five years of administration experience.

## 4. Research Measurements

### 4.1. FA Approach of Quantitative Analysis

Table 2 illustrates that the calculated numbers of the Kaiser–Meyer–Olkin measurement of sampling adequacy was 0.736, which means the questionnaire data from the 231 questionnaires were generally accepted for the measured equation of the FA of quantitative analysis, according to the commonly used Kaiser’s metrics of the Kaiser–Meyer–Olkin measurement. A measurement of 0.9 or more is very suitable; 0.8 is suitable; 0.7 is generally accepted; 0.6 is not suitable; below 0.5 is extremely unsuitable. Critically, the appraised numbers of significance of the Kaiser–Meyer–Olkin measure and Bartlett’s test was 0.000…, which is much lower than 0.05, which shows that the FA of quantitative analysis was apparently suitable for measuring the interactive communalities among these four appraised, five assessed and two evaluated criteria in the 231 valid interviewed questionnaires.

Furthermore, Table 3 demonstrates the commonalities among these four appraised, five assessed and two evaluated criteria in the 231 valid interviewed questionnaires in the FA of quantitative analysis. The commonalities of the SM-SPPDGRCL, OM-BNSW, RM-FACSDI and KM-EKTUU (appraised criterion—the public philosophy (PP) of the learning community model (LCM) for the socializationism of the social learning theory (SLT)) were 0.719, 0.729, 0.689 and 0.705. Continuously, the commonalities of Articles 3, 4, 5, 6 and 11 (assessed criterion—the pursuing excellence philosophy (PEP) of the learning community model (LCM) for the organizationism of the social learning theory (SLT)) were 0.752, 0.711, 0.704, 0.723 and 0.689. Ultimately, the commonalities of Articles 12 and 16 were (evaluated criterion—the democracy philosophy (DP) of the learning community model (LCM) for the individualism of the social learning theory (SLT)) were 0.775 and 0.769.

Particularly, the majority of the entire commonalities of the appraised, assessed and evaluated criteria were higher than 0.6 and close to 0.7, which means there was a higher associated interplay among each criterion through measuring the 231 valid large-scale questionnaires in the FA approach of quantitative analysis.

### 4.2. THM Measurements of Qualitative Analysis

After executing the FA of quantitative analysis, the entire communalities among these four appraised, five assessed and two evaluated criteria in the 231 valid interviewed questionnaires were integrated into the computation of Equations (2) and (3) of the TEM of qualitative analysis for achieving the higher research validity and representativeness. Furthermore, the 30 valid experts’ weighted questionnaires were conducted into Equations (2) and (3) of the THM of qualitative analysis through the statistic entropy method with the communalities of the FA of quantitative research. Table 4 describes the weighted measurements of the first 10 academic experts in the THM of qualitative analysis.

Continuously, Table 5 demonstrates the weighted measurements of the second 10 empirical experts using the THM of qualitative analysis.

Finally, Table 6 illustrates the weighted measurement of the third 10 governmental experts using the THM method of qualitative analysis.

### 4.3. Comprehensive Results Discussion

In view of Table 4, Table 5 and Table 6, the 10 academic experts, 10 empirical experts and 10 governmental experts of the professional questionnaires all deemed the SM-SPPDGRCL (assessed dimensions for the multilateral organization performance assessment network (MOPAN) of the Food and Agriculture Organization (FAO) of the United Nations (UN), the cognitive institutions role of the institutional theory (IT), public philosophy (PP) of the learning community model (LCM) and the socializationism of the social learning theory (SLT)) was affected by Article 12 (evaluated criterion—the regulative role of the institutional theory (IT), democracy philosophy (DP) of the learning community model (LCM) for the individualism of the social learning theory (SLT)) and Articles 3, 4 and 5 (assessed criterion—the normative role of the institutional theory (IT), pursuing excellence philosophy (PEP) of the learning community model (LCM) and the organizationism of the social learning theory (SLT)).

Continuously, they also agreed that the OM-BNSW (assessed dimensions for the multilateral organization performance assessment network (MOPAN) of the Food and Agriculture Organization (FAO) of the United Nations (UN), the cognitive institutions role of the institutional theory (IT), public philosophy (PP) of the learning community model (LCM) and the socializationism of the social learning theory (SLT)) was affected by Article 16 (evaluated criterion—the regulative role of the institutional theory (IT), democracy philosophy (DP) of the learning community model (LCM) for the individualism of the social learning theory (SLT)) and Article 11 (assessed criterion—the normative role of the institutional theory (IT), pursuing excellence philosophy (PEP) of the learning community model (LCM) and the organizationism of the social learning theory (SLT)).

## 5. Conclusions and Recommendations

Since the continuous appearances of a succession of the recent global FHAS incidents, a majority of people have commenced to realize that the FHAS is not only a governmental responsibility, but that they also have a public duty. Facing the development trend of the world’s FHAS, the Taiwanese government instituted the comprehensive 20 Articles of the TFAEL on 19 April 2022 for overcoming various current global food security challenges of the FAHS. In order to cross-evaluate the legitimacy, effectiveness, implemented performance, international compatibility and educational sustainability of the 20 Articles of the TFAEL, the three role considerations (normative, regulative and cognitive institutions) of the institutional theory (IT), the three essential philosophies of the learning community model (LCM) and the three basic characteristics (individualism, organizationism and socializationism) of the social learning theory (SLT) were cross-employed to measure the large-scale and experts’ weighted questionnaires through the factor analysis (FA) of quantitative analysis. As a result, the most two valuable conclusions and findings are concluded as follows:

(1) Not only Article 12 (evaluated criterion—the regulative role of the institutional theory (IT), democracy philosophy (DP) of the learning community model (LCM) for the individualism of the social learning theory (SLT)), but also Articles 3, 4 and 5 (assessed criterion—the normative role of the institutional theory (IT), pursuing excellence philosophy (PEP) of the learning community model (LCM) and the organizationism of the social learning theory (SLT)) can empirically facilitate the strategic management for supporting and promoting the TFHAS policy dialogue at the global, regional and country levels in order to effectively advance the international sustainable compatibility of the TFAEL. The TFHAS has instituted the source of law that the central, municipal and local levels of the Taiwanese government must set the TFHAS-related competent authorities, responsible agencies or departments to facilitate the planning, supervision, awards and professional labor training of the TFHAS. Particularly, the central, municipal and local governments also have the direct duty to organize the professional committees to facilitate the implementation, performance, development and gathering resources for the administrative guidance of the TFHAS.
(2) Article 16 (evaluated criterion—the regulative role of the institutional theory (IT), democracy philosophy (DP) of the learning community model (LCM) for the individualism of the social learning theory (SLT)) as well as Articles 6 and 11 (assessed criterion—the normative role of the institutional theory (IT), pursuing excellence philosophy (PEP) of the learning community model (LCM) and the organizationism of the social learning theory (SLT)) can completely construct the operational management for building normative and standard-setting TFHAS works in order to efficiently advance the international sustainable compatibility of the TFAEL. Each TFHAS-related competent authority, responsible agency or department has to guide cater and food businesses and industries to use local ingredients, label the origin of the ingredients according to the municipality, county (city) or township (town, city) name, and promote the consumption of local agricultural products, reduce food waste, reduce the amount of ingredients, and reduce leftovers by giving priority to domestic and local ingredients sourced in Taiwan.

Therefore, beyond the analytical results of this research, the international sustainable compatibility of the TFAEL is a direction towards sustainable food systems, food policies and agricultural food markets and food chains for overcoming the various contemporary global food security challenges. However, as for the limited research resources, there are more measured methodologies of the quantitative and qualitative analyses to be utilized to detect the key determinants of the evaluation and construct the assessed model in the future.

## Figures and Tables

**Table 1:** The description of statistics of factor analysis (FA) approach.

Gender	Male: 142 (61.48%)		Female: 89 (38.52%)	
Geography	Northern Taiwan ^1^: 65 (28.13%)	Middle Taiwan ^2^: 103 (34.58%)	Southern Taiwan ^3^: 51 (22.07%)	Eastern Taiwan ^4^: 12 (15.22%)
How many courses regarding the international sustainability practice of the TFHASE have you taken before?	0: 32 (13.85%)	1: 142 (61.47%)	2: 41 (17.75%)	3 and up to 3: 16 (6.93%)
Have you heard about the TFHASE before?		Yes: 217 (93.94%)		No: 14 (6.06%)
Have you known about the TFAEL before?		Yes: 221 (95.67%)		No: 10 (4.33%)
Will you support the international sustainability practice of the TFHASE through the TFAEL?		Yes: 174 (75.32%)		No: 57 (24.68%)

^1^: Chilung, Taipei, New Taipei and Taoyuan cities. ^2^: Hsinchu, Miaoli, Taichung and Changhua cities. ^3^: Yunlin, Chiayi, Tainan and Kaohsiung cities. ^4^: Hualien and Taitung counties.

**Table 2:** The description of statistics of FA approach.

Kaiser–Meyer–Olkin measure of sampling adequacy		0.736
	Chi-squared test	583.318
Bartlett’s test of sphericity	df	12
	Significance	0.000…

**Table 3:** The commonalities of KMO and Bartlett’s test of the FA approach of quantitative analysis.

Criteria	Initial	Extraction
SM-SPPDGRCL—Assessed dimensions for the multilateral organization performance assessment network (MOPAN) of the Food and Agriculture Organization (FAO) of the United Nations (UN) (the cognitive institutions role of the institutional theory (IT), public philosophy (PP) of the learning community model (LCM) and the socializationism of the social learning theory (SLT))	1	0.719
OM-BNSW—Assessed dimensions for the multilateral organization performance assessment network (MOPAN) of the Food and Agriculture Organization (FAO) of the United Nations (UN) (the cognitive institutions role of the institutional theory (IT), public philosophy (PP) of the learning community model (LCM) and the socializationism of the social learning theory (SLT))	1	0.729
RM-FACSDI—Assessed dimensions for the multilateral organization performance assessment network (MOPAN) of the Food and Agriculture Organization (FAO) of the United Nations (UN) (the cognitive institutions role of the institutional theory (IT), public philosophy (PP) of the learning community model (LCM) and the socializationism of the social learning theory (SLT))	1	0.689
KM-EKTUU—Assessed dimensions for the multilateral organization performance assessment network (MOPAN) of the Food and Agriculture Organization (FAO) of the United Nations (UN) (the cognitive institutions role of the institutional theory (IT), PP of the LCM and the socializationism of the social learning theory (SLT))	1	0.705
Article 3 (Assessed criterion—the normative role of the institutional theory (IT), pursuing excellence philosophy (PEP) of the learning community model (LCM) and the organizationism of the social learning theory (SLT))	1	0.752
Article 4 (Assessed criterion—the normative role of the institutional theory (IT), pursuing excellence philosophy (PEP) of the learning community model (LCM) and the organizationism of the social learning theory (SLT))	1	0.711
Article 5 (Assessed criterion—the normative role of the institutional theory (IT), pursuing excellence philosophy (PEP) of the learning community model (LCM) and the organizationism of the social learning theory (SLT))	1	0.704
Article 6 (Assessed criterion—the normative role of the institutional theory (IT), pursuing excellence philosophy (PEP) of the learning community model (LCM) and the organizationism of the social learning theory (SLT))	1	0.723
Article 11 (Assessed criterion—the normative role of the institutional theory (IT), pursuing excellence philosophy (PEP) of the learning community model (LCM) and the organizationism of the social learning theory (SLT))	1	0.689
Article 12 (Evaluated criterion—the regulative role of the institutional theory (IT), democracy philosophy (DP) of the learning community model (LCM) for the individualism of the social learning theory (SLT))	1	0.775
Article 16 (Evaluated criterion—the regulative role of the institutional theory (IT), democracy philosophy (DP) of the learning community model (LCM) for the individualism of the social learning theory (SLT))	1	0.769

**Table 4:** The first 10 academic experts’ weighted measurements of the THM of qualitative analysis.

Evaluated criterion—the regulative role of the institutional theory (IT), democracy philosophy (DP) of the learning community model (LCM) for the individualism of the social learning theory (SLT)		Assessed dimensions for the multilateral organization performance assessment network (MOPAN) of the Food and Agriculture Organization (FAO) of the United Nations (UN) (the cognitive institutions role of the institutional theory (IT), public philosophy (PP) of the learning community model (LCM) and the socializationism of the social learning theory (SLT))	Assessed criterion—the normative role of the institutional theory (IT), pursuing excellence philosophy (PEP) of the learning community model (LCM) and the organizationism of the social learning theory (SLT)				
Article 12 (0.705)	Article 16 (0.664)		Article 3 (0.752)	Article 4 (0.711)	Article 5 (0.704)	Article 6 (0.723)	Article 11 (0.689)
0.1915	0.1193	SM-SPPDGRCL (0.719)	0.1677	0.1419	0.146	0.076	0.0098
0.0988	0.1247	OM-BNSW (0.729)	0.0466	0.091	0.1338	0.1247	0.1214
0.1149	0.1157	RM-FACSDI (0.689)	0.1438	0.0696	0.0169	0.0638	0.0707
0.0693	0.0599	KM-EKTUU (0.705)	0.0598	0.0085	0.1323	0.1036	0.1085

**Table 5:** The second 10 empirical experts’ weighted measurements of the THM of qualitative analysis.

Evaluated criterion—the regulative role of the institutional theory (IT), democracy philosophy (DP) of the learning community model (LCM) for the individualism of the social learning theory (SLT)		Assessed dimensions for the multilateral organization performance assessment network (MOPAN) of the Food and Agriculture Organization (FAO) of the United Nations (UN) (the cognitive institutions role of the institutional theory (IT), public philosophy (PP) of the learning community model (LCM) and the socializationism of the social learning theory (SLT))	Assessed criterion—the normative role of the institutional theory (IT), pursuing excellence philosophy (PEP) of the learning community model (LCM) and the organizationism of the social learning theory (SLT)				
Article 12 (0.705)	Article 16 (0.664)		Article 3 (0.752)	Article 4 (0.711)	Article 5 (0.704)	Article 6 (0.723)	Article 11 (0.689)
0.1534	0.0909	SM-SPPDGRCL (0.719)	0.116	0.0154	0.1265	0.076	0.0634
0.078	0.1193	OM-BNSW (0.729)	0.0855	0.045	0.0901	0.1139	0.1994
0.0912	0.065	RM-FACSDI (0.689)	0.0987	0.0835	0.0228	0.0957	0.0833
0.0416	0.0019	KM-EKTUU (0.705)	0.1114	0.0243	0.124	0.0749	0.1214

**Table 6:** The third 10 governmental experts’ weighted measurements of the THM of qualitative analysis.

Evaluated criterion—the regulative role of the institutional theory (IT), democracy philosophy (DP) of the learning community model (LCM) for the individualism of the social learning theory (SLT)		Assessed dimensions for the multilateral organization performance assessment network (MOPAN) of the Food and Agriculture Organization (FAO) of the United Nations (UN) (the cognitive institutions role of the institutional theory (IT), public philosophy (PP) of the learning community model (LCM) and the socializationism of the social learning theory (SLT))	Assessed criterion—the normative role of the institutional theory (IT), pursuing excellence philosophy (PEP) of the learning community model (LCM) and the organizationism of the social learning theory (SLT)				
Article 12 (0.705)	Article 16 (0.664)		Article 3 (0.752)	Article 4 (0.711)	Article 5 (0.704)	Article 6 (0.723)	Article 11 (0.689)
0.1915	0.16	SM-SPPDGRCL (0.719)	0.1716	0.1076	0.1347	0.0468	0.0173
0.0954	0.0836	OM-BNSW (0.729)	0.1006	0.0668	0.0803	0.1486	0.1339
0.1149	0.0505	RM-FACSDI (0.689)	0.0811	0.023	0.0096	0.0729	0.0554
0.0536	0.0071	KM-EKTUU (0.705)	0.0679	0.0059	0.1159	0.0454	0.0678

## Data Availability

The data used to support the findings of this study can be made available by the corresponding author upon request.

## Abbreviations

FHAS	Food Health and Agriculture Safety
TFHAS	Taiwanese Food Health and Agriculture Safety Education
TFAEL	Taiwanese Food and Agricultural Education Law
SM-SPPDGRCL	Strategic Management for Supporting and Promoting the Taiwan Food Health and Agriculture Safety Policy Dialogue at Global, Regional and Country Levels
OM-BNSW	Operational Management for Building Normative and Standard-Setting TFHAS Works
RM-FACSDI	Relationship Management for Facilitating Food Health and Agriculture Safety, Advocacy, Communication and Sharing of Data and Information
KM-EKTUU	Knowledge Management for Encouraging Food Health and Agriculture Safety Knowledge and Technology Uptake and Update
